# Involvement of GPx-3 in the Reciprocal Control of Redox Metabolism in the Leukemic Niche

**DOI:** 10.3390/ijms21228584

**Published:** 2020-11-14

**Authors:** Christine Vignon, Christelle Debeissat, Jérôme Bourgeais, Nathalie Gallay, Farah Kouzi, Adrienne Anginot, Frédéric Picou, Philippe Guardiola, Elfi Ducrocq, Amélie Foucault, Noémie Ravalet, Louis-Romée Le Nail, Jorge Domenech, Marie-Christine Béné, Marie-Caroline Le Bousse-Kerdilès, Emmanuel Gyan, Olivier Herault

**Affiliations:** 1 CNRS ERL 7001 LNOx “Leukemic Niche & Redox Metabolism”, 37000 Tours, France; christine.vignon@gmail.com (C.V.); cdebeissat@yahoo.fr (C.D.); j.bourgeais@chu-tours.fr (J.B.); n.langonne@chu-tours.fr (N.G.); farah_kouzi@hotmail.com (F.K.); f.picou@chu-tours.fr (F.P.); ducrocq@med.univ-tours.fr (E.D.); amelie.foucault@univ-tours.fr (A.F.); noemie.ravalet@univ-tours.fr (N.R.); jordomen@gmail.com (J.D.); emmanuel.gyan@univ-tours.fr (E.G.); 2 EA 7501, Faculty of Medicine, Tours University, 37000 Tours, France; 3 Department of Biological Hematology, Tours University Hospital, 37000 Tours, France; 4 Inserm UMR-S-MD1197, Paul Brousse Hospital, Paris-Saclay University, 94800 Villejuif, France; adrienne.anginot@inserm.fr (A.A.); caroline.le-bousse-kerdiles@inserm.fr (M.-C.L.B.-K.); 5 Inserm U1160, EMiLy, Saint-Louis Research Institute, Paris University, 75010 Paris, France; 6 CNRS GDR3697 Micronit “Microenvironment of Tumor Niches”, 37000 Tours, France; 7 Department of Genomic Oncology, Angers University Hospital, 349100 Angers, France; phguardiola@chu-angers.fr; 8 FHU GOAL “Grand-Ouest Acute Leukemia”, Angers University Hospital, 49100 Angers, France; mariechristine.bene@chu-nantes.fr; 9 Department of Orthopaedic Surgery, Tours University Hospital, 37000 Tours, France; lrlenail@hotmail.com; 10 Department of Biological Hematology, Nantes University Hospital, 44000 Nantes, France; 11 Department of Hematology and Cell Therapy, Tours University Hospital, 37000 Tours, France

**Keywords:** leukemic cell, mesenchymal stromal cell, reactive oxygen species, microenvironment, GPx-3

## Abstract

The bone marrow (BM) microenvironment plays a crucial role in the development and progression of leukemia (AML). Intracellular reactive oxygen species (ROS) are involved in the regulation of the biology of leukemia-initiating cells, where the antioxidant enzyme GPx-3 could be involved as a determinant of cellular self-renewal. Little is known however about the role of the microenvironment in the control of the oxidative metabolism of AML cells. In the present study, a coculture model of BM mesenchymal stromal cells (MSCs) and AML cells (KG1a cell-line and primary BM blasts) was used to explore this metabolic pathway. MSC-contact, rather than culture with MSC-conditioned medium, decreases ROS levels and inhibits the Nrf-2 pathway through overexpression of GPx3 in AML cells. The decrease of ROS levels also inactivates p38MAPK and reduces the proliferation of AML cells. Conversely, contact with AML cells modifies MSCs in that they display an increased oxidative stress and Nrf-2 activation, together with a concomitant lowered expression of GPx-3. Altogether, these experiments suggest that a reciprocal control of oxidative metabolism is initiated by direct cell–cell contact between MSCs and AML cells. GPx-3 expression appears to play a crucial role in this cross-talk and could be involved in the regulation of leukemogenesis.

## 1. Introduction

Acute myeloblastic leukemia (AML) is the first tumoral syndrome for which cancer stem cells/leukemia initiating cells (LICs) have been described [1,2]. Leukemic cells establish interactions with their surrounding bone marrow (BM) microenvironment and LICs are potentially located in hematopoietic niches that protect them from conventional chemotherapy and trigger the frequently observed relapses [3]. Thus, a better knowledge of the role of niche cells in the biology of leukemic cells has clinical relevance.

Redox metabolism plays a critical role in normal and leukemic hematopoiesis. The microenvironment of healthy hematopoietic stem cells (HSC) is a complex network with numerous interacting stromal cell types, differentiated hematopoietic cells [4,5,6,7,8], soluble regulators [9,10], and nerve fiber connections [11,12] that regulate cell fate. Several studies have highlighted the significant role of oxidative metabolism in the self-renewal and differentiation of HSCs. Thus, the most primitive HSCs are reactive oxygen species (ROS)^low^, associated with a lesser activation of the p38 mitogen-activated protein kinase (MAPK) compared to their ROS^high^ more differentiated counterpart [13,14]. The nuclear factor-erythroid 2 p45-related factor 2 (Nrf2), a sensor of intracellular ROS levels, plays a pivotal role in the maintenance of a normal cellular redox status [15,16]. The low ROS level observed in HSCs is essential for the maintenance of their self-renewal and is controlled, among others, through members of the forkhead box O (FoxO) family and the tumor-suppressor protein ataxia telangiectasia mutated (ATM) [17,18,19]. Activation of p38MAPK by ROS induces in HSCs a loss of self-renewal [13,20]. The higher ROS levels observed in more differentiated hematopoietic cells are then involved in differentiation and maturation processes in a homeostasis context [21,22]. The importance of oxidative metabolism has also been demonstrated in leukemic hematopoiesis, where low levels of ROS characterize the compartment of LICs [23,24]. Of note, ROS overproduction by NADPH oxidases (NOX) in AML cells promotes blast proliferation [25]. Studying mouse primary leukemias induced by *Hoxa9*-*Meis1,* Sauvageau et al. have reported a GPx-3/ROS/p38 MAPK axis which controls the aggressiveness of leukemia. In this model, GPX3 overexpressing LICs display low ROS levels associated with an inactivation of p38 MAPK [23]. Moreover, the importance of *GPX3* in AML biology is highlighted by the fact that the same authors demonstrated that the highest levels of GPX3 in primary human AML cells are from patients with adverse prognosis, classically associated with a high frequency of LICs [26].

More broadly, when considering energy metabolism in hematopoietic cells, HSCs exhibit lower mitochondrial respiration and respiratory capacities than progenitors cells [27], which is essential for their maintenance and long-term function [28,29,30,31]. In addition, Ito et al. showed that mitophagy participates in their self-renewal by degrading defective mitochondria [32]. During cell differentiation, the energy demand increases, making it necessary to accelerate the mitochondrial metabolism which promotes entry into the cell cycle as well as differentiation [33], in particular via an increase in ROS levels [34]. Conversely to normal hematopoiesis, leukemic cells present a differential sensitivity to modulators of glycolysis, which is involved in the initiation and maintenance of leukemia [35], as well as in drug resistance [36,37]. Moreover, AML cells (bulk) have a higher mitochondrial mass and an increased oxygen consumption rate in comparison to normal hematopoietic progenitors. Interestingly, mitochondrial and oxidative phosphorylation system (OXPHOS) activities have been reported as major regulators of chemoresistance in leukemic cells [38]. Finally, a recent publication by the group of Tonks established for the first time the relationship between ROS production and glycolysis to promote the proliferation of leukemic cells via PFKFB3 (6-phosphofructo-2-kinase/fructose-2,6-biphosphatase 3 enzyme) overexpression [39].

The aim of this study was to characterize the GPx3/ROS/p38MAPK axis and associated molecular pathways in cell partners of the leukemic niche before chemotherapy. The spread of leukemic cells in different sites of hematopoiesis leads them to interact with an initially nonleukemic hematopoietic microenvironment, which will promote leukemic development within specific niches. Using a model of leukemic niche established by coculturing primary BM mesenchymal stromal cells (MSCs) and AML cells, we established that the BM-MSCs contact promotes in leukemic cells an overexpression of GPX3, a decrease in ROS levels, the cytoplasmic relocalization of Nrf2, and an inactivation of p38MAPK. The concomitant inhibition of their proliferation was associated with a reduction of their energy/redox metabolism. Interestingly, reverse effects were observed in BM-MSCs for which the contact with leukemic cells promotes a decrease in GPX3 expression, higher ROS levels, and nuclear relocalization of Nrf2.

## 2. Results

### 2.1. Primary Bone Marrow MSCs Reduce the Proliferation of Leukemic Cells

**Figure 1:**
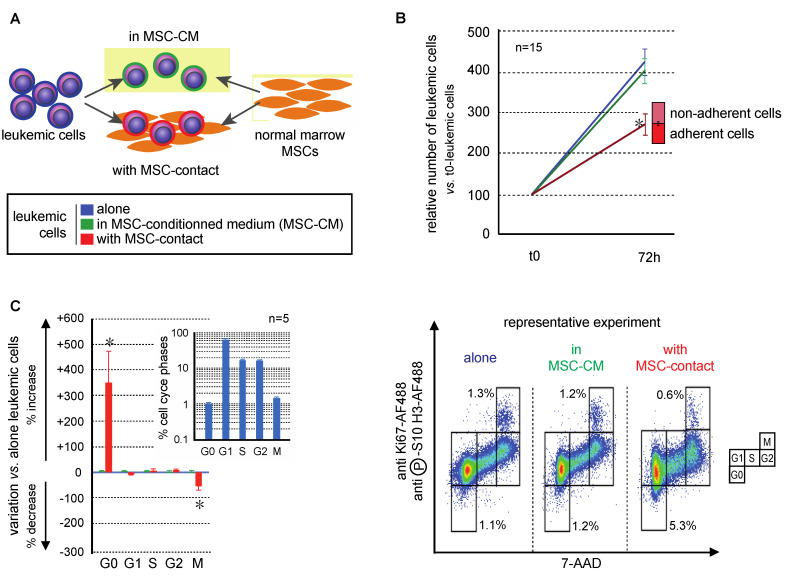
Mesenchymal stromal cells (MSCs) decreased leukemic cell proliferation. Experiments were performed with KG1a leukemic cells cultured alone (blue), with MSC-CM (green), or in coculture with MSCs (red). (**A**) Experimental design of mono- and coculture of MSCs and leukemic KG1a cells. Leukemic cells were cultured in medium alone, with MSC-CM or over MSCs; MSCs were cultured alone or with KG1a leukemic cells. (**B**) Leukemic cell growth was evaluated after 72 h of mono-, MSC-CM, or coculture (nonadherent cells represent cells that do not adhere to MSCs after 72 h of coculture, *n* = 15). (**C**) Cell cycle was analyzed by a flow cytometry multilabeling protocol using anti-Ki67-AF488, anti-phosphoS10-H3-AF488 and 7AAD (*n* = 5). Variations in MSC-CM and cocultured vs. monocultured KG1a in cell cycle phases after 72h are shown on the left and a representative experiment is presented on the right. * *p* < 0.05.

The effects of MSCs on the growth of leukemic cells of the AML KG1a cell-line were studied after 72h of culture in BM MSC-conditioned medium (MSC-CM) with or without contact with MSCs (Figure 1A). MSC-CM did not modify the growth of leukemic cells which was conversely was significantly decreased in the presence of MSCs in the culture system (Figure 1B; *p* < 0.0001). This indicates that MSC contact is necessary to control leukemia proliferation [40]. An original flow cytometry method was developed to precisely discriminate all the cell cycle phases and apoptosis of leukemic cells [41]. MSC-CM did not induce modifications in the cell cycle distribution of leukemic cells, while MSC-contact promoted their quiescence (G0 phase: 4.1 ± 0.7% vs. 1.0 ± 0.1%, *p* < 0.05) and decreased mitoses (M phase: 1.0 ± 0.1% vs. 1.5 ± 0.1%, *p* < 0.01), comparatively to controls (Figure 1C). Altogether, these results support an antiproliferative effect of MSCs on leukemic cells related to cell–cell interactions, without involvement of secreted factors.

**Figure 2:**
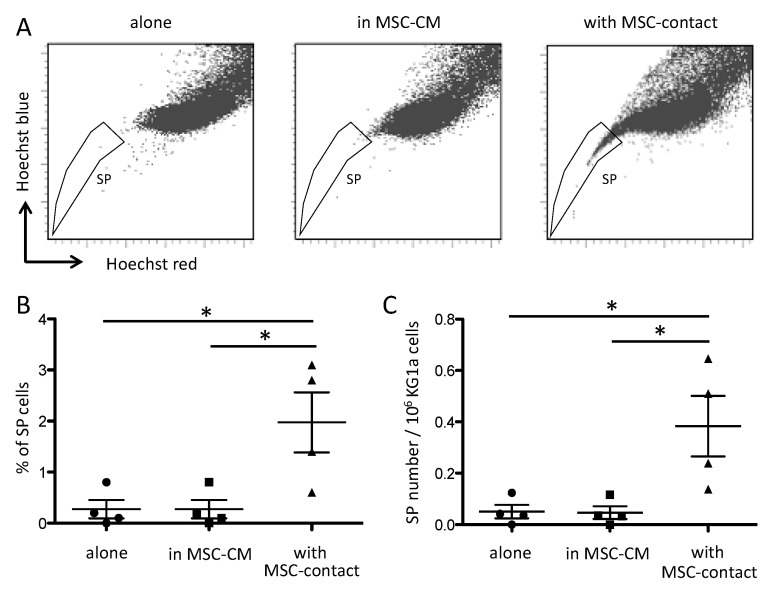
Contact with MSCs increases SP proportion in leukemic cells. SP was assessed by Hoechst efflux measurement in flow cytometry. Cytograms in (**A**) illustrate a representative acquisition of Hoechst staining of KG1a leukemic cells after a 72 h culture alone (left panel), in MSC-CM (middle panel), or with MSC-contact (right panel). Quantitative results are shown as SP percentages (**B**) absolute numbers (**C**) in the three culture conditions (*n* = 4). * *p* < 0.05.

It has been reported that MSCs modify the side population (SP) functionality of AML blasts [42]. As expected, leukemic cells cocultured with MSCs displayed a higher proportion of SP compared to cells cultured alone or with MSC-CM (Figure 2).

**Figure 3:**
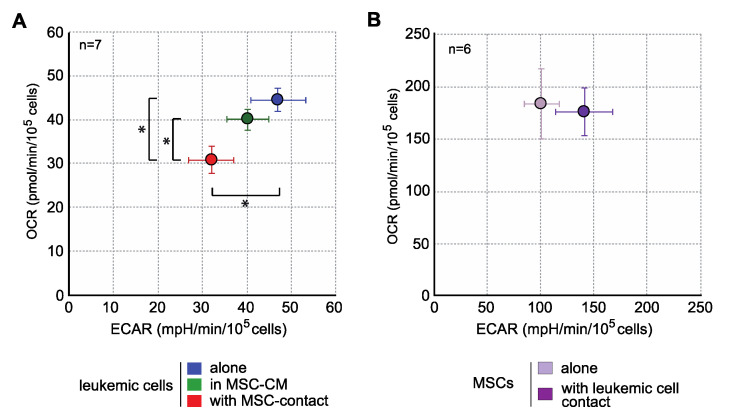
MSCs decrease the energy metabolism of leukemic cells. Energy metabolism was assessed through the evaluation of mitochondrial respiration (OCR) and glycolysis (ECAR) and with Seahorse XFe96 (*n* = 7). (**A**) Analysis of energy metabolism in KG1a cells. (**B**) Analysis of energy metabolism in MSCs. * *p* < 0.05.

Interestingly, when analyzing the energy metabolism by oxygen consumption rate (OCR) and extracellular acidification rate (ECAR) quantification, we observed that contact with MSCs induced an important decrease in the mitochondrial respiration and glycolysis of leukemic cells (Figure 3), without any metabolic impact of MSC-CM. This global metabolic inhibition is in line with the antiproliferative effect described above. In MSCs, leukemic cells induced a trend to a more glycolytic profile without modification of OCR (Figure 3). Global strategy and representative curves are shown in Figure S1.

### 2.2. MSCs and Leukemic Cells Reciprocally Interact to Modify Their Oxidative Metabolism

It has been well established that the proliferation of leukemic cells is associated with modifications of the redox metabolism [43]. Moreover, ROS regulate MSCs function and their ability to support hematopoiesis [44,45]. We were consequently interested in investigating redox metabolism by analyzing ROS levels and Nrf2 status in both mesenchymal and leukemic partners of the leukemic niche.

**Figure 4:**
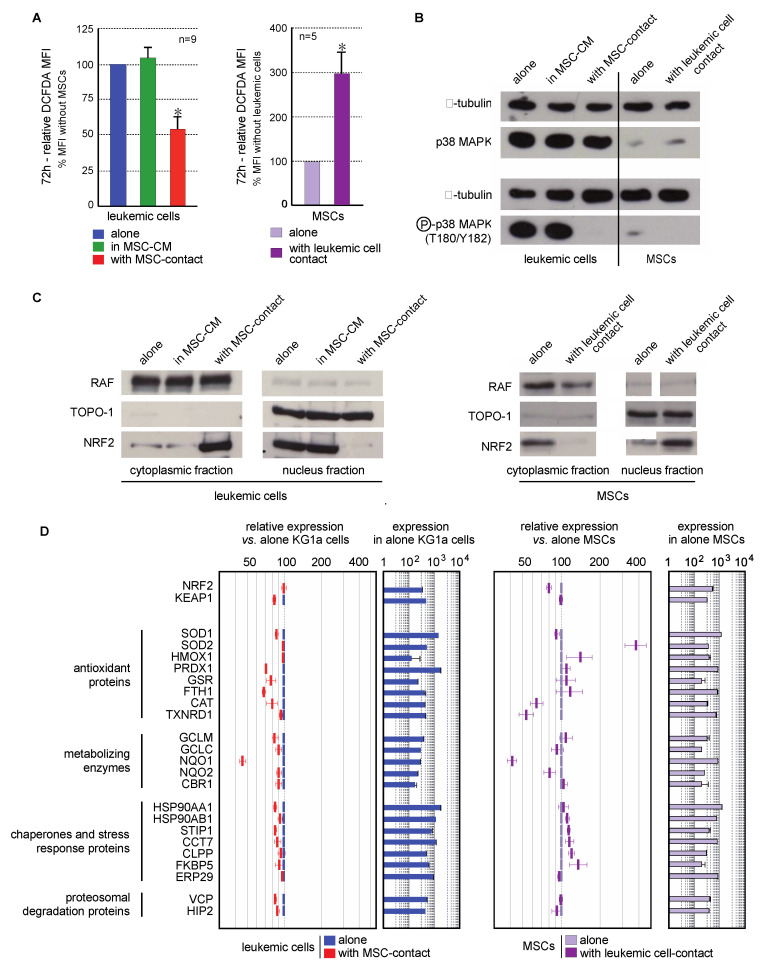
Interaction between leukemic cells and MSCs induces opposite oxidative metabolism and Nrf2 pathway modifications in both cell types. (**A**) Intracellular ROS level was analyzed by flow cytometry after CM-H_2_DCFDA staining in leukemic cells cultured alone (blue), with MSC-CM (green), or with MSCs (red) as well as in MSCs cultured alone (violet) or cocultured with KG1a cells (dark violet) for 72h (*n* = 5–9). (**B**) The expression and activation of p38MAPK were analyzed by Western blot in KG1a cultured alone, with MSC-CM or cocultured as well as in MSCs alone or cocultured with leukemic cells (*n* = 3); alpha-tubulin was used as loading control. (**C**) Nrf2 subcellular localization was analyzed by Western blot in the cytoplasmic and nuclear fractions of leukemic cells (left) or MSCs (right). RAF and TOPO-1 were used as loading controls and purity indicators of the cytoplasmic and nuclear fractions, respectively. (**D**) Nrf2 target genes expression was evaluated by transcriptomic analysis and is presented as relative expression vs. KG1a cells or MSCs alone (*n* = 3). * *p* < 0.05.

Contact with MSCs induced a decreased in ROS levels in leukemic cells (46.5 ± 8.9%, *p* < 0.001). This effect was not observed in culture with MSC-CM, or in nonadherent KG1a cells in coculture experiments, excluding a role of MSC-secreted factors in this antioxidant response (Figure 4A left). Interestingly, higher ROS levels (3-fold increase) were seen in MSCs cocultured with leukemic cells (Figure 4A right).

MAPK pathways are known to be activated by ROS, and more particularly p38MAPK, the inactivation of which is associated with self-renewal of normal and leukemic HSCs [13,20,23]. Consequently, p38MAPK activation (T180/Y182) was studied in line with the ROS status of cells in the MSC/leukemic cell coculture system. KG1a cells present a constitutive activation of p38MAPK that was abolished by contact with MSCs but not MSC-CM (Figure 4B). This is consistent with the reduced proliferation previously observed. Surprisingly, the oxidative stress promoted by leukemic cells in MSCs was not associated with an increased phosphorylation of p38MAPK.

The transcription factor Nrf2 is a cytoplasmic redox sensor translocated in the nucleus when intracellular ROS levels increase, thereby inducing the expression of genes involved in the control of oxidant homeostasis [46]. The subcellular localization of Nrf2 and the transcriptional expression of its target genes were analyzed. Nrf2 was preferentially located in the nucleus of leukemic cells (Figure 4C left), in accordance with their proliferating profile and ROS levels reported above. As expected, the antioxidant effect of MSCs on leukemic cells was concomitant with an inhibition of Nrf2-nuclear translocation (Figure 4C left). This cytoplasmic location was associated with a global underexpression of Nrf2 target genes (Figure 4D). Conversely to leukemic cells, MSCs spontaneously presented a Nrf2 cytoplasmic localization (Figure 4C right). Contact with leukemic cells favored the nuclear translocation of Nrf2 and the overexpression of numerous Nrf2-target genes (Figure 4D right), also in accordance with the increased ROS levels previously described.

Moreover, the quantification of ROS levels in nine stromal cell-lines showed that the lowest ROS levels were observed in MS5, AFT024 and 2018 (Figure S2), which are the three stromal cell-lines supporting normal hematopoiesis [47]. Altogether, primary MSCs promoted the quiescence of leukemic cells in which a decrease of ROS levels inactivates Nrf2 and p38MAPK pathways. Conversely, leukemic cells modified MSCs oxidative metabolism through an increase in ROS levels, which activates the Nrf2 pathway, highlighting a potential mechanism of bone marrow niche remodeling by leukemic cells.

### 2.3. GPx3 Expression is Inversely Regulated in Leukemic Niche Partners

**Figure 5:**
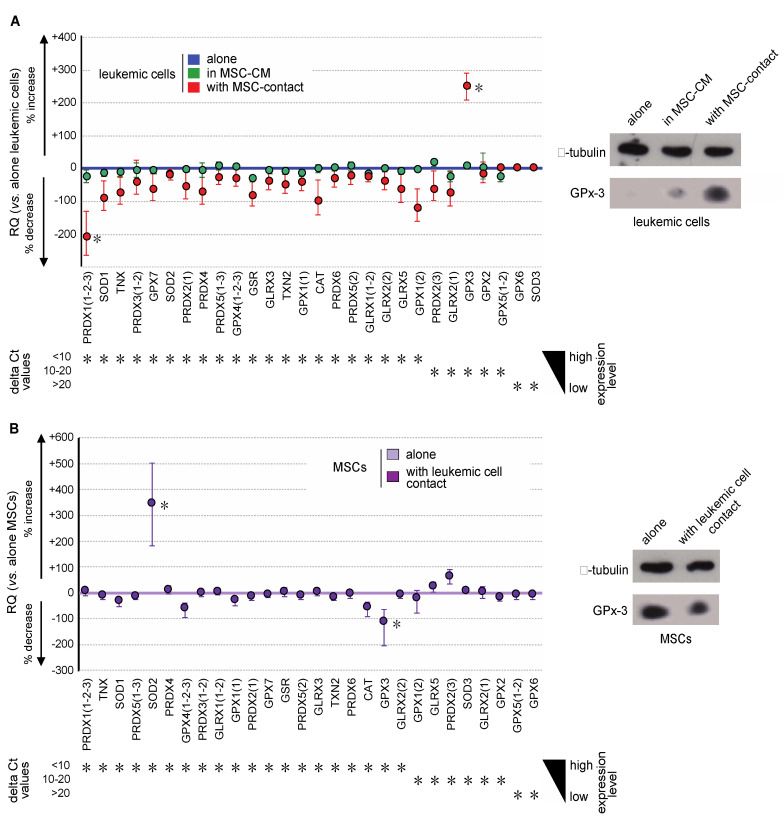
Leukemic cells and MSCs reciprocally modify their gene expression profile of antioxidant enzymes. (**A**) Gene expression of the major antioxidant enzymes was analyzed by real-time PCR in KG1a cells and is presented, from the highest to the lowest expressed genes in KG1a cells, as the percentage of increased or decreased relative expression (RQ = 2^−ΔΔ*C*t^) in KG1a cells cultured in MSC-CM (green dots) or cocultured with MSCs (red dots) vs. KG1a cells alone (blue line) (left). Increased GPX3 expression was studied at the protein level by Western blot analysis (right), alpha-tubulin being used as loading control. (**B**) Gene expression of major antioxidant enzymes was analyzed by real-time PCR in MSCs and is presented, from the highest to the lowest expressed gene in MSCs alone, as the percentage of increased or decreased relative expression (RQ = 2^−ΔΔ*C*t^) in cocultured MSCs (violet dots) vs. MSCs alone (violet line) (left). Decreased GPX3 expression was confirmed at the protein level by Western blot analysis (right), alpha-tubulin being used as loading control. * *p* < 0.05.

Oxidative metabolism results from a balance between ROS production and their degradation by enzymatic processes. To dissect more thoroughly the molecular mechanisms implied in the regulation of ROS among niche partners, the expression of major antioxidant genes (*SOD1*, *SOD2*, *SOD3*, *CAT*, *TXN*, *TXN2*, *GLRX*, *GLRX2*, *GLRX3*, *GLRX5*, *PRDX*, *PRDX2*, *PDRX3*, *PRDX4*, *PRDX5*, *PRDX6*, *GPX1*, *GPX2, GPX3*, *GPX4*, *GPX5*, *GPX6*, *GPX7,* and *GSR*) was evaluated in MSCs and leukemic cells. Transcriptional expression was quantified by real-time PCR in the different cellular fractions of the coculture system. Variations in gene expression were considered as biologically significant whenever at least a 2-fold change was observed. In accordance with the lack of variation in ROS levels, the expression of antioxidant genes in leukemic cells was not impacted by culture in MSC-CM (Figure 5A left). Conversely, contact with MSCs induced a trend to underexpression of all analyzed antioxidant genes, except *GPX3,* the expression of which was 3.4-fold increased (Figure 5A left). This overexpression was confirmed at the protein level by Western blot analysis (Figure 5A right). Conversely, Gpx-3 expression was significantly decreased in MSCs at both transcriptional and protein levels, upon stimulation by contact with leukemic cells (Figure 5B). In these cells, we observed a concomitant *SOD2* overexpression, which can be explained by the Nrf2 activation previously observed (Figure 4D), the sequence of *SOD2* containing the *NFE2L2* response element.

*GPX3* is known as a determinant of leukemic hematopoiesis [23] and its niche-induced overexpression in leukemic cells fits well with modifications in redox metabolism. Interestingly, among the nine stromal cell-lines studied, the highest level of GPX3 was found in MS-5 (Figure S1), the mesenchymal cell-line able to efficiently support hematopoiesis, pointing out GPx3 as an important regulator of oxidative metabolism in MSCs.

### 2.4. MSCs Modify ROS Levels, p38MAPK Activation, and GPX3 Expression in Primary AML Cells

The effects of MSCs on the *GPX3*/ROS/p38 MAPK axis in primary BM AML blasts were analyzed to confirm the results obtained with KG1a cells.

**Figure 6:**
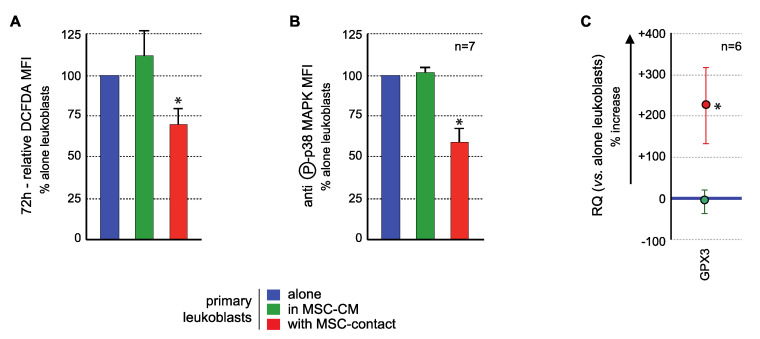
MSCs interact with primary AML cells to modify their ROS levels, p38MAPK activation, and GPX3 expression. Analysis was performed in primary AML cells cultured alone (blue), with MSC-CM (green) or with MSCs (red). (**A**) ROS levels were analyzed in primary AML cells by flow cytometry after CM-H_2_DCFDA labelling (*n* = 13 with MSC-CM; *n* = 26 with MSC-contact). Primary BM-blasts from 20 AML patients were studied by coculture experiments on normal BM-MSCs from five donors (26 coculture experiments were performed due to various combinations). Seventeen AML patients (among these 20 patients) presented a decrease in ROS levels upon contact with MSCs. Blasts were used no more than three times. As expected, when AML patient blasts were studied on different MSCs, the results were not different, ruling out any MSC batch effect. Whenever possible (sufficient number of blasts), the effect of CM-MSCs was evaluated (*n* = 13), and in all cases, CM-MSCs did not induce a decrease in ROS levels in AML blasts. All statistics were performed comparing different groups using the Kruskal–Wallis test comparing coculture condition (*n* = 26), CM-MSCs condition (*n* = 13) and control condition (blasts alone). (**B**) p38MAPK expression and activation were studied by flow cytometry after labelling with anti-p38 or anti-phospho (T180/Y182)-p38, respectively (*n* = 7); (**C**) *GPX3* expression was studied by real-time PCR. Results are presented as percentage of increased or decreased relative GPX3 quantity (RQ = 2^−ΔΔ^*^C^*^t^) in MSC-CM or MSCs cocultured AML cells vs. AML cells cultured alone (*n* = 6). * *p* < 0.05.

ROS levels analysis demonstrated a significant ROS reduction in blast cells in contact with MSCs compared to controls, in 17/20 AML patients tested, with a mean inhibition of 27.2 ± 5.4% (*p* < 0.005) Culture in MSC-CM had no significant effect on the ROS levels of primary leukemic cells (Figure 6A) nor of nonadherent primary blasts in coculture experiments. Flow cytometry analysis of p38 MAPK activation (T180/Y182 phosphorylation) showed that MSCs contact induced a decreased phosphorylation (mean fluorescence intensity (MFI): 4.2 ± 1.5 AU vs. 7.85 ± 1.87 AU in controls, *p* < 0.0005) while no effect was observed with MSC-CM (7.96 ± 1.57 AU) (Figure 6B) or in nonadherent primary blasts in coculture experiments. Transcriptional expression of *GPX3* was quantified in blast cells. While MSC-CM did not affect GPX3 level, contact with MSCs induced a significant 3.25 ± 0.94 mean fold increase (*p* < 0.05) of GPX3 level in primary leukemic cells (Figure 6C).

## 3. Discussion

BM mesenchymal stromal cells (MSCs) have been reported to regulate the level of ROS in normal HSCs [40], but their impact on redox metabolism in leukemia is poorly understood.

Oxidative metabolism is a key element in both normal and leukemic hematopoiesis. In the last decade, several studies have highlighted the essential role of oxidative metabolism not only in the tight regulation of normal HSCs [13,17,20] but also in LSC biology [23,24,48]. Thus, low ROS contents have been associated with stemness and quiescence [13,17,20,23] while intermediate ROS levels were found in more differentiated populations and associated to proliferation and differentiation [49]. The importance of the microenvironment has also been emphasized, not only in the maintenance of normal hematopoiesis homeostasis [10,50,51] but also in leukemic development [52]. In this study, we used a coculture system composed of primary MSCs from healthy donors and AML cells (cell-lines or primary blasts) to model the leukemic niche and dissect molecular pathways reciprocally induced in the field of redox metabolism.

In this coculture model, a decrease in leukemic cell growth was demonstrated to be associated to a reduced cell cycle when blasts were in contact with MSCs but not with MSC-CM, supporting an antiproliferative effect of MSCs on leukemic cells related to cell–cell interactions, without involvement of secreted factors. This inhibition has already been observed in different leukemias [53,54]. In addition, an increased proportion of leukemic cells expressing an SP phenotype was observed when in contact with MSCs. These results are in agreement with those by Boutin et al. who reported SP phenotype induction and ABC transporter activation in AML blasts in contact with MSCs, thereby conferring chemoresistance [42]. We also observed an inhibition of mitochondrial respiration and glycolysis in leukemic cells in contact with MSCs, indicating a global energy metabolism reduction, consistent with the reduced proliferation observed. Thus, the microenvironment induces a protective effect that favors quiescence, low energy metabolism, and overall less active leukemic cells.

Analysis of the redox status showed that contact with MSCs induces a drop of ROS levels in the KG1a cell-line and in primary AML cells, associated with a decreased activation of p38MAPK, in line with previous data describing ROS as inducers of the MAPK pathways [20]. As expected in this lower ROS context, leukemic cells in contact with MSCs mostly localize Nrf-2 in the cytoplasm, thereby in its nonactive form. Consequently, Nrf-2 target genes were found underexpressed compared to controls. The expression profile of the principal antioxidant genes was globally decreased, except for an overexpression of *GPX3* that we already described as important in murine LIC biology [23]. The transcriptional increase of *GPX3* in KG1a cells and primary AML blasts was confirmed at the protein level in KG1a cells. The group of Mendez-Ferrer et al. recently published a relationship between the chemoresistance of leukemic cells to AraC and MSC-induced modifications of energy/redox metabolism. Interestingly, *GPX3* was overexpressed in treated leukemic cells cocultured with MSCs [55]. Altogether these data reinforce the identification of a key role of the BM microenvironment in the control of the GPx3-ROS-p38MAPK axis in leukemia and of the oxidative metabolism of leukemic blasts. Upstream determinants/inducers involved in this axis need to be elucidated.

**Figure 7:**
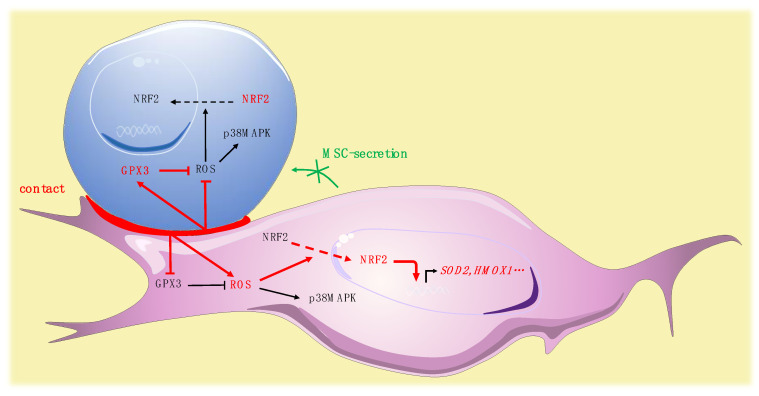
Pivotal role for Gpx-3 in ROS regulation and oxidative metabolism in leukemic niche (leukemic cell in blue, MSC in purple).

We simultaneously analyzed the effect of the contact of leukemic cells on MSCs. In MSCs, leukemic cells induced a trend to a more glycolytic profile without modification of OCR, in accordance with the glycolytic switch described in the tumor microenvironment [56]. Interestingly, leukemic cells induced a rise of ROS in MSCs. This modification in the redox status of MSCs may impact their functionality, since a defective support of normal hematopoiesis by MSCs stimulated by leukemic cells has been reported [57]. Our group recently highlighted a specific profile of connexins, which are components of gap junctions, in leukemic MSCs [58]. The reversed ROS kinetics, namely drop in leukemic cells and rise in MSCs, may be partly explained by a previously described mechanism of ROS transfer between hematopoietic cells and MSCs by Connexin-43 [59]. The specific role of connexins, particularly connexin-43, should be further explored. Surprisingly, the oxidative stress promoted by leukemic cells in MSCs was not associated with an increased phosphorylation of p38MAPK. Independent regulation of p38MAPK activation has nonetheless already been described in the context of oxidative stress, potentially explained by early ROS-induced phosphatase activation [60]. Nrf-2, in MSCs, is predominantly located in the cytoplasm and contact with leukemic cells modifies its subcellular localization, inducing a strong nuclear translocation. Surprisingly, analysis of the expression of Nrf-2 target genes revealed a balanced profile, with overexpression of half of the target genes tested and underexpression of *NQO1*, *TXNRD1,* and *CAT*. The molecular profile of antioxidant enzymes showed that leukemic cells increased the expression of *SOD2* while inhibiting the expression of *GPX3*. Altogether, these data underline the impact of leukemic cells on MSC oxidative metabolism, suggest a pivotal role for Gpx-3 in this process and reinforce previous work reporting remodeling of the bone marrow microenvironment by leukemic cells [61] (Figure 7).

In conclusion, the BM microenvironment plays a pivotal role in leukemia occurrence and chemoresistance. The development of new therapeutic strategies requires a better understanding of the reciprocal interplay evidenced here and of the pathways activated downstream. This study identifies that BM MSC-contact modulates the oxidative metabolism in leukemic cells, involving the regulation of Nrf-2 pathway, overexpression of GPx3, and inhibition of p38MAPK. Reciprocally, leukemic cells modify their microenvironment by inducing oxidative stress and consecutive activation of the Nrf-2 pathway in MSCs. The GPx-3-induced ROS decrease in leukemic cells, already reported in LICs [23], concomitantly associated with the antiproliferative effect of MSC contact, reinforces the therapeutic interest of targeting leukemic niche interactions to limit the niche-induced chemoresistance in AML.

## 4. Materials and Methods

### 4.1. Cells

The human acute myeloid leukemia cell-line KG1a (FAB M0/M1, CD34^+^) was purchased from the European Collection of Authenticated Cell Cultures (ECACC, Wiltshire, UK) and cultured in Minimum Essential Medium Alpha (αMEM, Gibco BRL, ThermoFisher Scientific, Waltham, MA, USA) supplemented with 10% heat-inactivated fetal bovine serum (FBS, HyClone), 2 mM L-glutamine (Gibco BRL), 100 IU/mL penicillin, and 100 µg/mL streptomycin (Gibco BRL). Cells were maintained at 37 °C and 5% CO_2_ in a humidified atmosphere.

Primary human leukemic cells from AML patients at diagnosis were collected after informed consent for cell banking and following a procedure approved by the ethical committee of Tours University Hospital (CPP Tours identifier ID-RCB: 2011-A00262-39/approval date: 7 July 2011). Mononuclear cells were isolated following Ficoll density gradient centrifugation (20 min, 400× *g*), frozen in Iscove medium supplemented with 10% FBS and 10% DMSO, and stored in liquid nitrogen until use.

Human bone marrow (BM) MSCs were isolated from healthy volunteers after informed consent and following a procedure approved by the ethical committee of Tours University Hospital (CPP Tours identifier ID-RCB: 2016-A00571-50/approval date: 7 June 2016). Cells were amplified as described previously [62]. Briefly, total bone marrow cells were seeded at 5 × 10^4^ cells/cm^2^ in αMEM (Gibco BRL) supplemented with 10% FBS (Hyclone, GE Healthcare, Chicago, IL, USA), 2 mM glutamine (Gibco BRL), 100 IU/mL penicillin, 100 µg/mL streptomycin, and 1 ng/mL FGF-2. Adherent cells were cultured until 90% confluence and frozen (P0) in SVF-10% DMSO. The adipogenic, chondrogenic, and osteogenic differentiation capacities of BM-MSCs were verified (Figure S3) as described previously [62].

### 4.2. KG1a and Primary Blast Cell Cocultures

Primary P0 BM-MSCs isolated from healthy patients were thawed and cultured at P1 in αMEM (-) supplemented with 10% FBS, 2 mM glutamine, 100 IU/mL penicillin, 100 µg/mL streptomycin, and 1 ng/mL FGF-2. Cells were trypsinized at 90% confluence, seeded at P2 at 5–8 × 10^4^ cells/cm^2^, and cultured for 4–6 days until near confluence.

For KG1a experiments, MSC culture medium (MSC-CM) without FGF-2 was renewed on the day before the experiment. At T0 1.5 × 10^4^ KG1a cells/cm^2^ were seeded in medium alone, in MSC-CM (24 h-supernatant of BM-MSCs) or on primary BM-MSCs. After 72 h, nonadherent cells were harvested with phosphate buffered saline (PBS). Adherent cells were trypsinized with PBS/EDTA 1mM for protein expression study and with trypsine/EDTA for other studies. Cocultured cells were sorted by fluorescence-activated cell sorting (FACS) for RNA analysis or by magnetic procedure for protein analysis.

For experiments on primary blasts, the MSC culture medium was changed with X-Vivo10 (Lonza, Basel, Switzerland) supplemented with 10% FBS on the day before the experiment. At T0, primary blast cells were thawed gradually in HBSS-2mM EDTA-100U/mL DNase (Roche Diagnostic, Basel, Switzerland), washed in HBSS-2 mM EDTA, and seeded in X-Vivo10 supplemented with 10% FBS, 10 ng/mL hIL-3 (ImmunoTools GmbH, Friesoythe, Germany), 10 ng/mL hFlt-3L (Immunotools), 10 ng/mL hTPO (Peprotech, Neuilly-Sur-Seine, France), and 20 ng/mL rhSCF (Immunotools) at 1.5 × 10^4^ cells/cm^2^, alone, with MSC-CM or over MSCs. After 72 h of culture, cells were analyzed directly by flow cytometry for phospho (T180/Y182)-p38, p38, and ROS expression or sorted by magnetic procedure for *GPX3* transcriptional analysis, as described thereafter. Flow cytometry strategies for analyses of leukemic and BM-MSCs are presented in Figure S4.

### 4.3. Fluorescence Activated Cell Sorting

Cells were incubated for 20 min at room temperature (RT) with allophycocyanin (APC) conjugated anti-CD45 IgG1 (J33) (Beckman Coulter, Brea, CA, USA), and fluorescein isothiocyanate (FITC) conjugated anti-CD90 IgG1 (5E10) (BD Biosciences, Franklin Lakes, NJ, USA) or appropriate isotype controls. Cells were washed with PBS and sorted by FACS MoFlo (Beckman Coulter) in culture medium supplemented with 10% heat inactivated FBS. After one wash with PBS, cells were resuspended in 1 mL Trizol^®^ (Invitrogen, ThermoFisher Scientific, Waltham, MA, USA) and stored at −80 °C.

For the most accurate sorting we chose the “purified mode” and a droplet envelope of one drop. The sorting speed was around 5000 cells/s.

### 4.4. Immuno-Magnetic Selection

Cells were labelled using the EasySep^TM^ PE positive selection kit (Stem Cell Technologies, Vancouver, Canada) according to the manufacturer’s instructions. Cells were incubated for 15 min at RT with anti-human CD32 blocker and anti-CD45-PE (eBiosciences, ThermoFisher Scientific, Waltham, MA, USA) or anti-CD90-RPE (Dakocytomation, Agilent Technologies, Santa Clara, CA, USA), then 15 min at RT with the EasySep PE selection cocktail, and finally for 10 min at RT with EasySep magnetic nanoparticles. For KG1a cells, coculture fractions were separated with an EasySep magnet. An adapted protocol was necessary for primary human leukemic cells. Labelled primary human leukemic cells were washed with PBS-2% SVF-2mM EDTA and separated on LS and then MS columns (Miltenyi Biotec, Bergisch Gladbach, Germany) according to the manufacturer’s instructions. Cell purity of both fractions was over 99.5%.

### 4.5. Flow Cytometric Analysis of Cell Cycle

Detailed cell cycle analysis of KG1a cells in each condition was performed by quantifying G0, G1, S, G2, and M phases as previously reported [28]. For quantification, 10^6^ cells were permeabilized with 1mL of ice cold ethanol (1 h, 4 °C), washed twice with PBS, 1% FBS, 0.25% Triton X-100 (PFT), and stained in 200 µL of PBS-FBS-TritonX100 (PFT) for 30 min at RT in the dark with 1 µg of 7-aminoactinomycin D (7-AAD, Sigma-Aldrich, St. Louis, MO, USA), 5 µL of Alexa Fluor^®^488-conjugated anti-human Ki67 mAb (B56) (BD Biosciences), and 2 µL of Alexa Fluor^®^488-conjugated anti-phospho(ser10)-histone H3 polyclonal antibody (Cell Signaling Technology, Danvers, MA, USA), respectively. After two washes with PFT, cells were stained with 10 µL of APC-Cy7-conjugated anti-CD45 (BD Biosciences) for 15 min at 4 °C. Cells were then washed twice with PBS and acquired on a FACS Canto II. Data analysis was performed with FlowJo software (v10.5.3, FlowJo, Ashland, OR, USA).

### 4.6. SP Detection

Hoechst staining was performed as previously described [29]. After a 72 h culture, cells were counted and resuspended at 10^6^ cells/mL in prewarmed (37 °C) Dulbecco’s modified Eagle’s medium containing 2% FCS/10 mM HEPES/Hoechst 33,342 (final concentration: 5 mg/10^6^ cells/mL) and incubated at 37 °C for 90 min under delicate shaking. After incubation, cells were placed on ice and stained with anti-CD45 antibody and a viability dye during 20 min on ice and at dark. Cells were then washed and analyzed on a BD Fortessa apparatus with Diva software (BD Biosciences).

### 4.7. Oxygen Consumption Rate (OCR) and Extracellular Acidification Rate (ECAR) Analysis

Leukemic cells were selected through a magnetic procedure. Cellular oxygen consumption rate (OCR) and extracellular acidification rate (ECAR) data were obtained using a Seahorse XF96 Flux analyzer (Agilent Technologies) as previously reported [44]. Experiments were performed according to the manufacturer’s instructions. The XF96 sensor cartridges were hydrated with 200 µL of Calibrant (Seahorse Bioscience, Agilent Technologies, Santa Clara, CA, USA) at pH 7.4 and stored overnight at 37 °C without CO_2_. Leukemic cells were seeded in poly-D-Lysine coated XF96 cell culture plates at 100,000 cells per well in XF Base medium supplemented with glutamine (2 mM) (pH 7.4). Cells were then incubated at 37 °C in a non-CO_2_ incubator for 1h and measurements were performed in the Seahorse XF96 Flux analyzer. Sequential injection of glucose (10 mM), oligomycin (1 µM), carbonilcyanide p-triflouromethoxyphenylhydrazone (FCCP; 3 µM), and rotenone/Antimycine A (0.5 µM) was performed according to the supplier’s technical specifications and permitted the determination of the principal metabolic parameters.

### 4.8. Analysis of Intracellular H_2_O_2_

ROS levels were quantified by staining cells with 2′,7′-dichlorodihydrofluorescein diacetate, acetyl ester (H_2_DCFDA, Invitrogen). Cells were washed twice with X-Vivo10 and incubated with APC-conjugated anti-human CD45 IgG1 (J33) (Beckman Coulter) or APCeFluor780 conjugated anti-human CD45 (eBiosciences) and 9.6 µM H_2_DCF-DA at 37 °C for 10 min. Cells were washed in cold PBS and immediately analyzed with a FACS Canto II instrument (BD Biosciences). Data analysis was performed with FlowJo software (v10.5.3).

### 4.9. Expression of Antioxidant Genes

Analyses were performed by real-time PCR on the LightCycler^®^ 480 microwell plate-based cycler platform (Roche Applied Science, Basel, Switzerland) using Universal ProbeLibrary assays designed with the ProbeFinder software (Roche Applied Science), as previously described [63]. RNA from each sample were reverse transcribed using the SuperScript^®^VILO^TM^ cDNA Synthesis kit (Invitrogen) according to the manufacturer’s instructions. Primers were purchased from Invitrogen and Universal ProbeLibrary probes from Roche Applied Science (nucleotide sequences of the primers and probes on request). Real-time PCR reactions were carried out in a total volume of 10 μL using LightCycler^®^ 480 Probes Master (Roche Applied Science) with a program of 10 min at 95 °C followed by 45 cycles of 10 s at 95 °C, 30 s at 60 °C, and a final cooling step of 10 s at 40 °C. All reactions were run in triplicate, and average values were used for quantification. Antioxidant gene expression was determined comparatively to the reference gene *GAPDH.* The relative antioxidant gene expression was analyzed using the 2^−ΔΔ*C*t^ method [64].

### 4.10. Protein Assays

Cells were lysed with Cytobuster^TM^ Protein Extraction Reagent (EMD Biosciences, Gibbstown, NJ, USA) supplemented with 0.7% protease inhibitor cocktail, 0.3% phosphatase inhibitor cocktail 1, 0.3% phosphatase inhibitor cocktail 2 (Sigma-Aldrich). To block GPx-3 protein secretion, 10 µg/mL Brefeldin A (Sigma-Aldrich) was added for the last 5 h of coculture.

For subcellular protein fractionation, cells were washed in PBS, resuspended in lysis buffer A containing KCl 10 mM, EDTA 1 mM, Hepes 20 mM, Glycerol 10%, NP40 0.2%, PMSF 10 µM, DTT 1 mM, protease inhibitor cocktail (Sigma-Aldrich), 0.3% phosphatase inhibitor cocktail (Sigma Aldrich), and incubated 5 min on ice. The lysed suspension was centrifuged 2 min at 12,000 *g* and cytoplasmic proteins included in supernatants were collected and kept at −80 °C until use. Pellets were resuspended in lysis buffer B containing NaCl 0.4 M, Glycerol 20%, Hepes 20 mM, KCl 10 mM, EDTA 1 mM, %, PMSF 10 µM, DTT 1 mM, protease inhibitor cocktail, 0.3% phosphatase inhibitor cocktail, incubated 30 min at +4 °C, and centrifuged 5 min at 20,000× *g*. Supernatants containing nuclear proteins were saved and kept at −80 °C until use.

Protein quantification was performed by a BCA protein assay (Sigma-Aldrich).

Aliquots of protein extracts (20 µg) were loaded onto 10% Next Gel (Amresco^®^, Interchim, Montluçon, France) and transferred to PVDF membranes. GPx-3 polyclonal antibody (1/200; Novus Biologicals, Centennial, CO, USA), p38 MAPK, and phospho-p38 MAPK (T180/Y182) monoclonal antibodies (1/1000; Cell Signaling), Nrf2 antibody (R&D systems, Minneapolis, MN, USA) or alpha-tubulin monoclonal antibody (1/1000; Cell Signaling) were blotted overnight at +4 °C. Secondary antibodies conjugated with HRP were incubated for 45 min at RT. The blots were revealed with Immun-Star^TM^ HRP Chemiluminescent Kit (BioRad, Hercules, CA, USA).

### 4.11. p38/Phospho (T180/Y182)-p38 Analysis by Flow Cytometry

Cells were fixed for 30 min at RT in 3.7% formaldehyde (Sigma Aldrich)-0.03% saponin (Sigma Aldrich), washed twice in PBS-10% FBS-0.03% saponin, and incubated for 1 h with anti-p38 (1/200; Cell signaling) or anti-phospho (T180/Y182)-p38 (1/200; Cell Signaling), washed in PBS-10% FBS-0.03% saponin, and then incubated for 30 min with goat anti-rabbit IgG Alexa Fluor 488 (Molecular Probes, ThermoFisher Scientific, Waltham, MA, USA) at 1/1000. Cells were washed with PBS before flow cytometry. Sample acquisition was performed with a FACS Canto II instrument. Data analysis was performed with FlowJo software (v10.5.3).

### 4.12. Statistics

All data are presented as mean ± standard deviation of 3 to 25 independent experiments. Nonparametric tests (Mann–Whitney or Kruskall–Wallis tests followed by Dunn post-hoc test) were used for statistical analyses. Statistical significance of real-time PCR data was analyzed with the Friedmann test followed by a Nemenyi post hoc test. Statistical analysis was performed on R (v3.3.1, www.r-project.org).

## Supplementary Materials

Supplementary Materials can be found at https://www.mdpi.com/1422-0067/21/22/8584/s1; Figure S1. Seahorse assay; Figure S2. ROS levels in stromal cell-lines; ROS levels were analyzed by flow cytometry after CM-H2DCFDA staining of MS5, AFT024, 2018, SR4987, BMC10, CCL226, BMC9, BFC012, and TrHBMEC cell-lines; Figure S3. Adipogenic, chondrogenic, and osteogenic differentiation capacities of BM-MSCs; Figure S4. Flow cytometry strategies for analyses of leukemic and BM-MSCs.

## Abbreviations

7-AAD	7-Aminoactinomycin D
AML	Acute myeloid leukemia
ATM	Ataxia telangiectasia mutated
BCA	Bicinchoninic acid
BM	Bone marrow
CAT	Catalase
CD	Cluster of differentiation
CFSE	Carboxyfluorescein succinimidyl ester
CM-H_2_DCFDA	5-(and-6)-chloromethyl-2′,7′-dichlorodihydrofluorescein diacetate, acetyl ester
DMSO	Dimethylsulfoxyde
DTT	Dithiothreitol
ECAR	Extracellular acidification rate
EDTA	Ethylenediaminetetraacetic acid
FACS	Fluorescence activated cell sorting
FBS	Fetal bovine serum
FCCP	Carbonilcyanide p-triflouromethoxyphenylhydrazone
FGF-2	Fibroblast growth factor-2
GAPDH	Glyceraldehyde 3 phosphate dehydrogenase
GLRX	Glutaredoxin
GPX	Glutathione peroxidase
GSR	Glutathione reductase
HBSS	Hank’s balanced salt solution
HSC	Hematopoietic stem cell
IL	Interleukin
LIC	Leukemia initiating cell
MAPK	Mitogen activated protein kinase
MEM	Minimum essential medium
MFI	Mean fluorescence intensity
MSC	Mesenchymal stromal cell
MSC-CM	Mesenchymal stem cell-conditioned medium
NOX	NADPH oxidase
Nrf2	Nf-E2 related factor 2
OCR	Oxygen consumption rate
PCR	Polymerase chain reaction
PFT	PBS-FBS-TritonX100
PMSF	Phenylmethylsulfonyl fluoride
PRDX	Peroxiredoxin
RAF	Rapidly accelerated fibrosarcoma
ROS	Reactive oxygen species
RQ	Relative quantification
RT	Room temperature
SCF	Stem cell factor
SOD	Superoxide dismutase
SP	Side population
TOPO-1	Topoisomerase-1
TPO	Thrombopoietin
TXN	Thioredoxin
