# Supplementary material for: Involvement of GPx-3 in the Reciprocal Control of Redox Metabolism in the Leukemic Niche
Source: Int J Mol Sci. 2020 Nov 14;21(22):8584. doi: 10.3390/ijms21228584 (PMC7696155; doi:10.3390/ijms21228584)
Supplement: Supplementary file 1 [file ijms-21-08584-s001.zip › Figure S3.pdf]

differentiation capacities of bone marrow MSCs

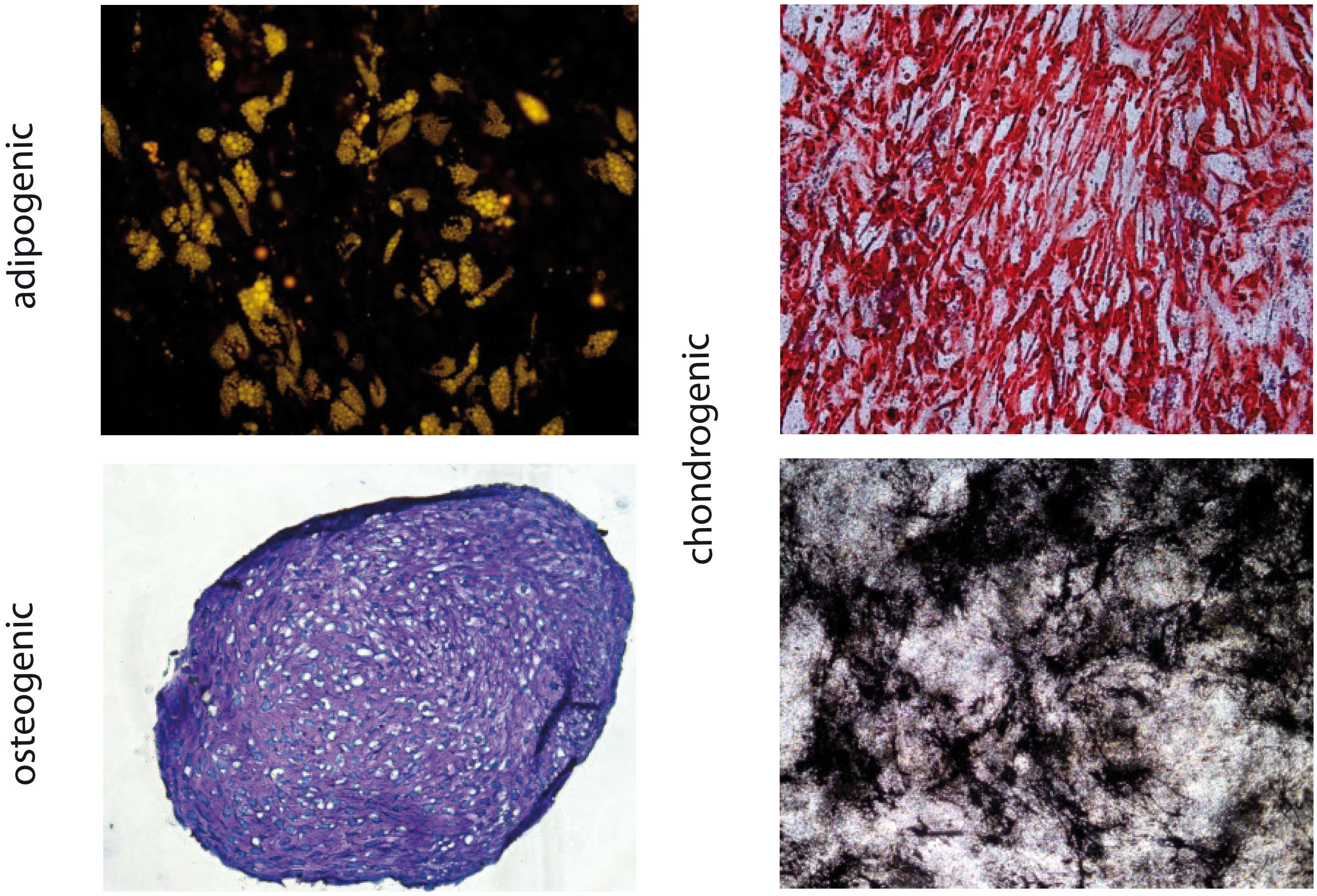

Figure S3
