# Supplementary figures and images for: Involvement of GPx-3 in the Reciprocal Control of Redox Metabolism in the Leukemic Niche
Source: Int J Mol Sci. 2020 Nov 14;21(22):8584. doi: 10.3390/ijms21228584 (PMC7696155; doi:10.3390/ijms21228584)

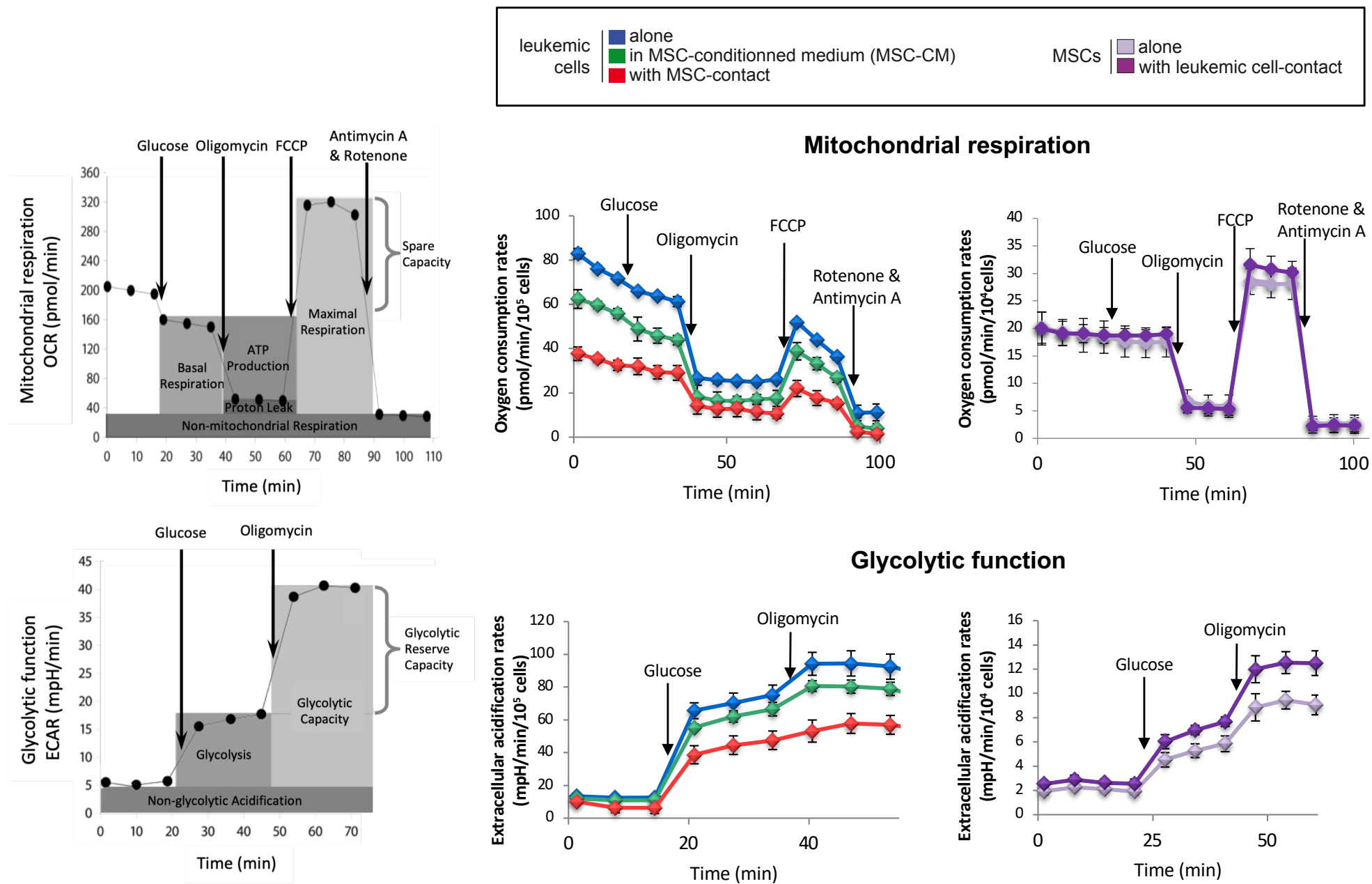

Figure S1

Supplement: Supplementary file 1 [file ijms-21-08584-s001.zip › Figure S1.pdf]

## ROS levels in stromal cell lines

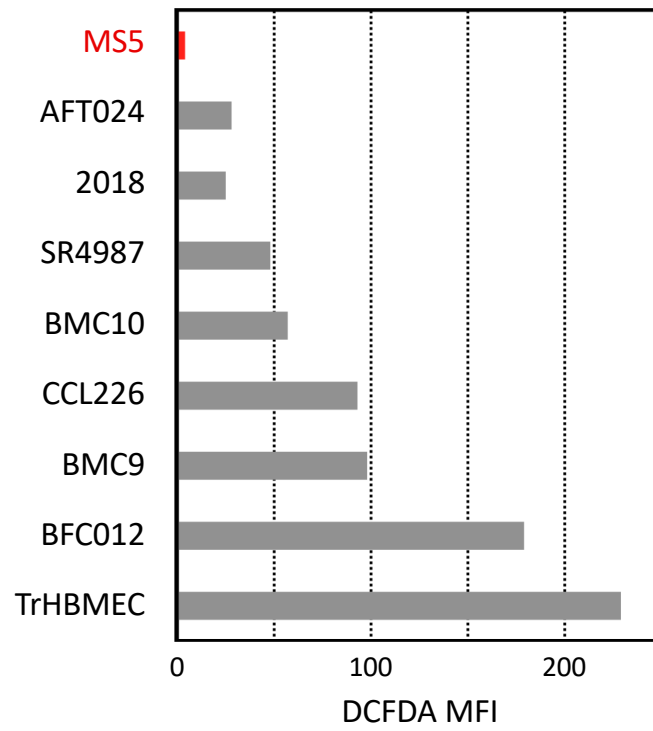

Figure S2

Supplement: Supplementary file 1 [file ijms-21-08584-s001.zip › Figure S2.pdf]

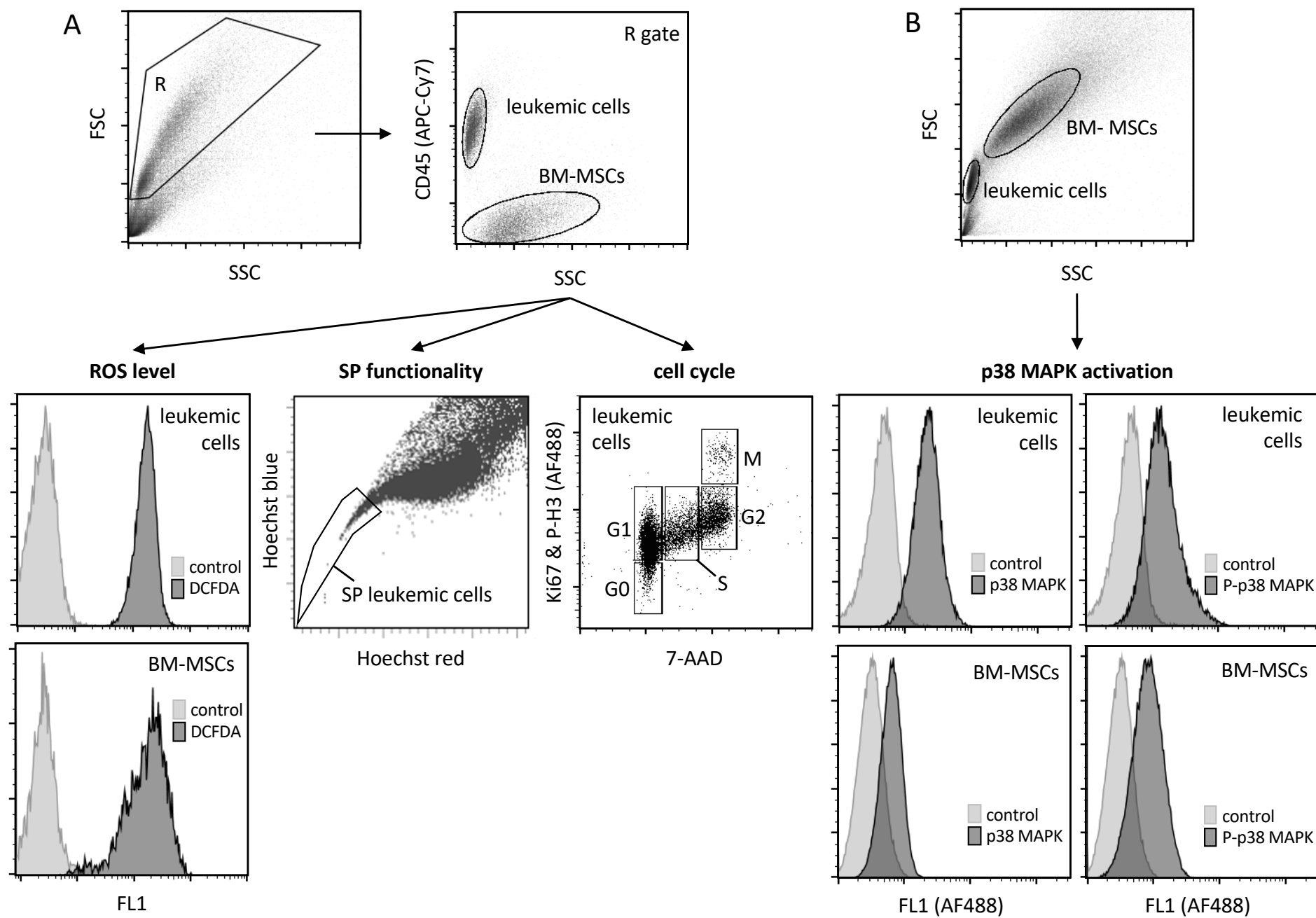

Figure S4

Supplement: Supplementary file 1 [file ijms-21-08584-s001.zip › Figure S4.pdf]
